# The role of environmental influences in the complex relationship between borderline personality disorder and attention-deficit/hyperactivity disorder: review of recent findings

**DOI:** 10.1186/s40479-019-0118-z

**Published:** 2020-01-06

**Authors:** Natalia Calvo, Benjamin Lara, Laia Serrat, Violeta Pérez-Rodríguez, Òscar Andión, Josep A. Ramos-Quiroga, Marc Ferrer

**Affiliations:** 1 BPD Program, Psychiatry Department, Hospital Universitary Vall d’Hebron, Centro de Investigación Biomédica en Red de Salud Mental (CIBERSAM), Barcelona, Spain; 2 Psychiatry and Legal Medicine Department, Universitat Autònoma de Barcelona (UAB), Barcelona, Spain; 3 Psychiatry, Mental Health and Addictions Group, Vall d’Hebron Institut de Recerca (VHIR), Barcelona, Spain; 4 Grup TLP Barcelona (BPD Barcelona Group), Barcelona, Spain

**Keywords:** Borderline personality disorder (BPD), Attention-deficit/hyperactivity disorder (ADHD), Childhood trauma, Abuse and neglect, Environmental influences, Emotion dysregulation, Impulsiveness

## Abstract

**Background:**

In recent years, the existence of possible developmental pathways from childhood Attention-Deficit/Hyperactivity Disorder (ADHD) to adult Borderline Personality Disorder (BPD) has been suggested. The existence of common genetic factors has been described but there is little evidence on the role of environmental factors in the possible transition from one disorder to another throughout life. The main goal of this work is to review the literature about the existing evidence on childhood traumas as factors that mediate the risk of developing BPD in children with ADHD.

**Methods:**

A literature search was conducted using PubMed, Science Direct and PsychInfo databases. Criteria included studies of BPD and ADHD relationships and childhood traumas as environmental influences from epidemiological or clinical samples.

**Results:**

The review only identified 4 studies that matched the search criteria. All studies retrospectively analyzed childhood traumas, and adult patients with BPD, with or without comorbid ADHD, were the most frequently mentioned. The analyzed evidence reinforces the relationship between the number of childhood traumas and higher clinical severity. Three of these analyzed studies describe an increased the risk of children with ADHD who report emotional and sexual traumatic experiences to develop BPD in adulthood.

**Conclusions:**

The experience of traumatic childhood events, especially those of an emotional type, may have a mediating effect of an increased risk of developing adult BPD in childhood ADHD patients. However, to consider them as risk factors, more studies, and especially longitudinal studies, are necessary to clarify the probable transactional process between the two disorders. Evidence from these studies may be helpful to develop early intervention programs to reduce the functional impairment associated with the two disorders.

## Background

Borderline Personality Disorder (BPD) is characterized by an enduring pattern of instability in interpersonal relationships, self-image and affect, and marked impulsivity [1]. DSM-5 also characterizes its development and course as a pattern of chronic instability, notably in early adulthood, with the greatest impairment found in the young-adult years. BPD is estimated to affect 1.6 to 5.9% of the adult general population [1]. Furthermore, the cumulative prevalence rates suggest that 1.4% of young people will meet diagnostic criteria for BPD by age 16 years, rising to 3.2% by age 22 [2]. Attention-Deficit/Hyperactivity Disorder (ADHD) is a common neurodevelopmental disorder emerging in childhood or early adolescence. ADHD is characterized by a pervasive pattern of developmentally inappropriate levels of inattention and/or hyperactivity-impulsivity that lead to clinically significant functional and psychosocial impairment [1]. In the general population, ADHD is estimated to affect about 5% of children and adolescents [1] and between 2.5–4% of the adults [3].

Different studies have highlighted the remarkable overlap of symptoms of BPD and ADHD in adulthood, as is the case of impulsiveness, which is considered a core component both of ADHD and BPD [4]. Emotional dysregulation (ED) is a set of symptoms that have been considered a core clinical feature of BPD and have been assigned a key role in the main etiopathogenic models of the disorder [5]. However, in recent years, the number of studies highlighting the relevant role that ED can also play in ADHD [6, 7] has increased. For differential diagnosis, suicidal behavior has been considered more typical of BPD than of ADHD [8], whereas primary attentional cognitive deficits are significantly more frequent in ADHD than in BPD [8, 9]. However, the diagnostic study in adults often concludes that both disorders can be diagnosed simultaneously [10], estimating a prevalence of BPD among adults with ADHD ranging from 19 to 37% [11] and of comorbid ADHD of 16–38% in patients with BPD as a primary diagnosis [12–14].

However, although previous studies have described this significant association between BPD and ADHD, its nature has not yet been fully clarified. The evidence suggests that the high frequency with which the two disorders coexist should be interpreted not only as a coincidence of two disorders at a certain period of life [15]. In this sense, the presence of ED in ADHD has been associated with worse functional impairment [12] and with a greater risk of suicidal behavior among young adults [7]. Similarly, compared to BPD without a comorbid ADHD, there is evidence of the fact that adult patients who can be simultaneously diagnosed with both disorders exhibit greater clinical severity with higher impulsivity and number of suicide attempts, and there is a greater likelihood of detecting other comorbid disorders, especially Substance Use Disorder (SUD) [12]. From the evidence of the interaction that can be established between the two disorders throughout life, possible pathways of childhood ADHD to adult BPD have been proposed, and earlier ADHD has been considered as a possible precursor of a later BPD [13, 14, 16, 17].

To study the transition of childhood ADHD to adult BPD, with or without comorbid ADHD, it is necessary to analyze the etiology of each disorder. From twins and twin family studies, ADHD heritability has been estimated to be between 60 and 80% [18], and around 40% for BPD [19]. Similar genetic findings, especially those involving the serotonergic and dopaminergic systems that have been associated with impulsivity and emotional dysregulation have been described in BPD and ADHD [19]. However, the phenotypic correlation between ADHD and Borderline Personality (BP) symptoms was explained not only by genetic factors (49%) but also by environmental factors (51%) [19]. It can therefore be hypothesized that the risk of developing BPD from childhood ADHD may be increased by possible common genetic etiologic factors, but the mediating effect that certain environmental events may have in this transition should also be taken into account. The most widely studied environmental variables as possible etiological factors of psychiatric disorders are childhood maltreatment, especially in Personality Disorders (PD) [20]. In the specific case of BPD, the experience of traumatic events in childhood, especially those that can compromise emotional maturation, has been considered as a predisposing factor for the later expression of ED and impulsivity, which are considered relevant clinical components of BPD [5, 21]. The relevance of childhood traumas in the development of ADHD is minor and has been associated mainly with cases that manifest ED [4, 22].

Thus, to analyze the factors that may have a mediating effect on the increase of the risk of developing BPD in childhood ADHD, it is advisable to study the variables that are more characteristic of the development of BPD than of ADHD. This would justify the main aim of this study, which is to analyze the literature about the existing evidence on the environmental factors involved in the developmental trajectory from childhood ADHD to adult BPD, namely traumatic experiences.

## Methods

In this paper, we performed a search for studies dealing with the association ADHD-BPD and environmental traumatic influences. For this purpose, the following bibliographic databases were searched: PubMed, Science Direct and PsychInfo. The following search terms were used: Borderline Personality Disorder AND Attention-Deficit/Hyperactivity Disorder AND childhood traumas or childhood maltreatment OR environmental vulnerability influences. Given the limited results, we added key terms by including emotional dysregulation, impulsivity, environmental, childhood, adolescence, in the association BPD and ADHD, and also in each of the disorders separately. As this is a review of recently published articles on the subject, the studies published before 2000, studies that were not published in English, unsystematic clinical case reports and treatment studies were excluded.

We included studies that explicitly mentioned the key terms. The titles and abstracts were screened to eliminate non-relevant and duplicate studies. When a title or abstract seemed to describe a study suitable for inclusion, the full-text article was obtained and examined to evaluate its relevance for our work. A total of 11 articles were potentially relevant in relation to the involvement of traumatic environmental factors in the transition from ADHD in childhood to BPD in adulthood. However, only 4 of them specifically and directly analyzed the possible contribution of traumatic childhood experiences in the evolution of one disorder to the other [14, 23–25] (see Table 1).

**Table 1:** Studies included in the revision with relevant information about the possible role of childhood traumas in the relationship between childhood Attention-Deficit/Hyperactivity Disorder (ADHD) and adult Borderline Personality Disorder (BPD)

Study	Size and election sample	Type of study	Targeted sample	Healthy control group	Assessment instrument	Main findings
Philipsen et al. 2008 [14]	BPD outpatients *N* = 118 women	Cross-sectional	Clinical study	No	WURS ADHD-CL SCID-I IPDE BSL CTQ	A significant association is described between childhood ADHD, childhood emotional abuse, and severity symptoms in women with BPD. Childhood ADHD may be considered a risk factor that predisposes to BPD development in the adulthood.
Prada et al. 2014 [24]	Outpatients *N* = 114 ADHD *N* = 99 BPD *N* = 67 BPD-ADHD	Cross-sectional	Clinical study	*N* = 415	SCID-II WURS ASRS v.11 CTQ BIS-10 STAXI LHA BDI-II BHS WHOQOL-BREF QFS	Compared with adult ADHD, adult BPD patients showed a higher number of childhood trauma.
Ferrer et al. 2017 [23]	Outpatients *N* = 82 BPD *N* = 22 ADHD *N* = 56 BPD-ADHD *N* = 44 No BPD-No ADHD	Cross-sectional	Clinical study	No	SCID-II DIB-R CTQ-SF CADDID WURS	Adult BPD patients with a comorbid ADHD presented the highest number of childhood trauma experiences. Childhood emotional and sexual abuse was associated to BPD diagnosis in the adulthood, with and without comorbid ADHD. The risk of developing an adult BPD in childhood ADHD could be associated to specific early traumas.
Dalbudak and Evren, 2015 [25]	Volunteer university students *N* = 271	Cross-sectional	Non-clinical study	No	BPI ASRS CTQ-28 BDI BAI	Depressive symptoms, emotional and physical abuse and severity of ADHD symptoms in adulthood were associated to severity of borderline personality features (BPF). These variables may play a mediator role on the relationship between childhood ADHD and adult BPF.

## Results

Differences in childhood trauma history between adult patients diagnosed with BPD, ADHD, and comorbid BPD-ADHD were assessed from the study of Prada et al., who analyzed the clinical differences between these disorders in adulthood [24]. This study used the Childhood Trauma Questionnaire (CTQ) [26, 27] for retrospective assessment of five types of maltreatment in infancy –emotional, physical and sexual abuse, and emotional and physical neglect. Compared to the group of healthy controls, the overall prevalence of childhood trauma history was higher in the clinical groups, especially in the BPD-ADHD group. The largest difference in prevalence between clinical groups and healthy controls was found in the concrete trauma of emotional abuse. Within the clinical groups, the only difference was in the rate of childhood sexual abuse, which was more frequent both in the BPD and the BPD-ADHD groups, compared to the ADHD group.

Ferrer et al.’s study [23] analyzed differences in childhood trauma history between adult BPD, ADHD and comorbid BPD-ADHD patients. Unlike Prada et al. [24], no healthy control group was included, but there was a clinical group of patients with BPD criteria, but without reaching the diagnostic threshold (group referred to as ‘non-BPD-no ADHD’). The assessment of childhood trauma history was also performed retrospectively using the CTQ [26, 27]. A greater total number of traumatic childhood events was described in BPD-ADHD patients, the most common being emotional and sexual traumas. In the specific comparison between ADHD and BPD-ADHD groups, the latter had a greater history of physical and emotional neglect. The authors found no differences in the number and type of traumatic antecedents between ADHD patients and ‘no BPD-no ADHD’ patients.

A study conducted with a non-clinical sample of university students was also analyzed [25]. Dalbudak and Evren studied the relationship between the history of traumatic childhood events, also assessed with the CTQ [26, 27], and the presence of ADHD symptomatology in adults and of BP features. A positive correlation was observed between the reference to the history of childhood emotional and physical abuse and the scores of the Adult ADHD Self-Report Scales (ASRS-v1.1) [28] and of the Borderline Personality Inventory (BPI) [29].

Not all analyzed studies have conducted analyses to estimate the effect of childhood traumas on the increased risk of developing a BPD in ADHD patients. At the clinical level, the study of Philipsen et al. [14] analyzed for the first time a possible association between the 5 types of maltreatment included in the CTQ [26, 27], BPD symptoms and childhood ADHD through a multivariate logistic regression analysis in a sample of 118 outpatient adult women diagnosed with BPD and BPD-ADHD. The results indicated a significant association between a history of emotional abuse in childhood, childhood ADHD and greater severity of BPD symptoms. In Ferrer et al.’s study [23], logistic regression analysis was also performed to explore a possible increased risk of developing adult BPD, ADHD or BPD-ADHD in patients of the study who reported childhood traumas. The results showed that reporting childhood emotional and sexual traumas was associated with the diagnosis of BPD in adulthood, with or without ADHD comorbidity. Also in non-clinical population [25], an association was described between a history of emotional and physical abuse in childhood, the severity of ADHD measured with the ASRS-v1.1, depressive symptoms measured with the Beck Depression Inventory (BDI) [30] and the BPI score [25].

## Discussion

Although the importance of trauma in the subsequent development of BPD has been extensively studied, the available evidence so far of the potential mediating role of childhood maltreatment in the evolution from childhood ADHD to adult BPD is very scarce. While most of the identified studies analyze childhood traumas as a secondary objective, the authors end up highlighting the relevance of the outcome of this specific analysis in the interpretation of the relationship between childhood ADHD and adult BPD.

Firstly, all the studies refer to the relationship between the experience of childhood trauma and the severity of psychopathology in adulthood. The study of Philipsen et al. [14] already proposed that emotional abuse in childhood was associated with greater severity, both of childhood ADHD and of adult BPD. In this sense, studies of Prada et al. [24] and Ferrer et al. [23] show the highest number of references to traumatic childhood antecedents in adult patients with BPD and comorbid ADHD. These findings are remarkable, as BPD-ADHD has been considered a severe type of BPD with a higher impulsive profile and more comorbid disorders [14, 23, 24], and severe traumatic childhood antecedents in BPD patients predict a worse response to the treatment [31]. In addition, the study of Dalbudak and Evren [25] describes the association between traumatic childhood antecedents and more ADHD symptoms and more BP features in non-clinical population. Although no reference is made to the functional impact associated with these clinical traits, this finding reinforces the evidence that traumatic childhood experiences may play a mediating role between early ADHD and the severity of later BPD.

Secondly, the descriptive analysis of the differences between the groups included in the studies of this review shows that most of the adult patients studied reported traumatic antecedents in their childhood, even those that did not reach the diagnostic threshold but suffered from functional impairment as a result of their psychopathology [23, 24]. Although the study of Prada et al. [24] observed differences in the prevalence of childhood trauma between healthy control versus clinical groups, similarly and more specifically, differences were found between the clinical groups, with a greater number of emotional and/or sexual abuses in BPD patients, with and without comorbid ADHD, in comparison with ADHD patients [23, 24]. These results indicate that, although severe forms of childhood maltreatment are present in multiple psychiatric disorders [32], there may be differences in prevalence even between disorders with very similar phenotypes such as BPD and ADHD. These results invite the question of whether the type of childhood trauma that has been associated with the development of BPD [5, 21, 33] may also increase the risk of this disorder in patients with childhood ADHD.

In relation to the analysis of the traumatic childhood event as a risk factor for a subsequent development of BPD in ADHD patients, a history of childhood sexual and emotional abuse has been associated with adult BPD or BPD-ADHD diagnosis [23], especially in cases with severe childhood ADHD [14]. In contrast, childhood physical trauma has been linked to an increased risk of ADHD persistence in adulthood [23]. Also in non-clinical population, traumatic childhood emotional abuse has been proposed as a risk factor for developing ADHD and BP features in adulthood [25]. Therefore, even with the limited evidence available, it seems that the experience of emotional abuse in children diagnosed with ADHD may be a mediating factor that increases the risk of subsequent development of BPD in adulthood. ED has been indicated as a mediator between a history of childhood maltreatment and high impulsivity in women with BPD but not in ADHD [21]. Previously, the mediating effect of ED had been described in the relationship between childhood ADHD and adult BPD in a sample of adult women diagnosed with BPD [34]. In short, as mentioned, children with more severe ADHD could be at higher risk of experiencing trauma and developing a later BPD [4, 14, 35]. In this developmental pathway, emotional trauma seems to favor ED and this, in turn, mediates subsequent development of BPD.

Despite the above-mentioned considerations, several limitations of the review should be noted. Despite the importance of these issues, to date, the studies carried out are limited, and we must be cautious when generalizing the results obtained. In addition, the type of studies from which this evidence comes have relevant methodological limitations, especially the fact that most of them are based on retrospective evaluations and there are no longitudinal studies. Therefore, the results may be conditioned by memory and recall bias and by patients’ subjective evaluations, with a high risk of overvaluing or undervaluing, and thereby reducing the reliability due to the type of designs. However, the fact that the different studies used the same diagnostic instrument, the CTQ, allows us to underline the results obtained. Nevertheless, future longitudinal studies would be necessary to assess the possible impact of trauma from childhood to adulthood from a developmental perspective for a better understanding of ADHD-BPD across the lifespan. Therefore, in the coming years, it is essential for studies to be carried out to define the biological and environmental risk factors to intervene early in childhood and adolescence, to improve their prognosis and to prevent the crystallization of these disorders.

## Conclusion

Although trauma has been extensively studied in relation to the development of BPD, in recent years, research has raised the question of its role in the ADHD-BPD association. This work reviews the studies published to date and notes that, although they are scarce, some conclusions can be drawn from the available evidence. They all agree that whether or not adult BPD develops from childhood ADHD could be conditioned by the differences in the environmental factors. Specifically, it can be hypothesized that exposure to childhood traumas, especially emotional trauma, which compromise the individual’s emotional maturation, could be one of the variables that increase the possibility of a child with ADHD developing BPD in adult life. The possibility of identifying the role of these variables can be highly beneficial for early diagnosis and intervention in these patients.

## Data Availability

As this paper does not present original data, this does not apply.

## Abbreviations

ADHD: Attention-Deficit/Hyperactivity Disorder
BPD: Borderline Personality Disorder
DSM: Diagnostic and Statistical Manual of Mental Disorders
ED: Emotional dysregulation
SUDs: Substance Use Disorders
